# ^1^H NMR Study of the HCa_2_Nb_3_O_10_ Photocatalyst with Different Hydration Levels

**DOI:** 10.3390/molecules26195943

**Published:** 2021-09-30

**Authors:** Marina G. Shelyapina, Oleg I. Silyukov, Elizaveta A. Andronova, Denis Y. Nefedov, Anastasiia O. Antonenko, Alexander Missyul, Sergei A. Kurnosenko, Irina A. Zvereva

**Affiliations:** 1 Faculty of Physics, Saint Petersburg State University, 7/9 Universitetskaya nab., 199034 Saint Petersburg, Russia; st064749@student.spbu.ru (E.A.A.); d.nefedov@spbu.ru (D.Y.N.); a.antonenko@spbu.ru (A.O.A.); 2 Institute of Chemistry, Saint Petersburg State University, 7/9 Universitetskaya nab., 199034 Saint Petersburg, Russia; oleg.silyukov@spbu.ru (O.I.S.); st040572@student.spbu.ru (S.A.K.); irina.zvereva@spbu.ru (I.A.Z.); 3 CELLS-ALBA Synchrotron, 08290 Cerdanyola del Vallès, Barcelona, Spain; amissiul@cells.es

**Keywords:** layered perovskite-like niobate, Dion-Jacobson phase, proton NMR

## Abstract

The photocatalytic activity of layered perovskite-like oxides in water splitting reaction is dependent on the hydration level and species located in the interlayer slab: simple or complex cations as well as hydrogen-bonded or non-hydrogen-bonded H_2_O. To study proton localization and dynamics in the HCa_2_Nb_3_O_10_·*y*H_2_O photocatalyst with different hydration levels (hydrated—α-form, dehydrated—*γ*-form, and intermediate—β-form), complementary Nuclear Magnetic Resonance (NMR) techniques were applied. ^1^H Magic Angle Spinning NMR evidences the presence of different proton containing species in the interlayer slab depending on the hydration level. For α-form, HCa_2_Nb_3_O_10_·1.6H_2_O, ^1^H MAS NMR spectra reveal H_3_O^+^. Its molecular motion parameters were determined from ^1^H spin-lattice relaxation time in the rotating frame (*T*_1ρ_) using the Kohlrausch-Williams-Watts (KWW) correlation function with stretching exponent β = 0.28: $E_{a}=0.210\left(2\right)$ eV, $\tau_{0}=9.0\left(1\right)\;\times \;10^{-12}$ s. For the β-form, HCa_2_Nb_3_O_10_·0.8H_2_O, the only ^1^H NMR line is the result of an exchange between lattice and non-hydrogen-bonded water protons. *T*_1ρ_(1/*T*) indicates the presence of two characteristic points (224 and 176 K), at which proton dynamics change. The γ-form, HCa_2_Nb_3_O_10_·0.1H_2_O, contains bulk water and interlayer H^+^ in regular sites. ^1^H NMR spectra suggest two inequivalent cation positions. The parameters of the proton motion, found within the KWW model, are as follows: $E_{a}=0.217\left(8\right)$ eV, $\tau_{0}=8.2\left(9\right)\;\times \;10^{-10}$ s.

## 1. Introduction

In recent years, layered perovskite-like oxides have attracted much attention because of their outstanding physical and chemical properties, including high-temperature superconductivity [1,2], colossal magnetoresistance [3], the capability of photocatalytic water decomposition under sunlight irradiation for further hydrogen storage [4,5], and ionic conductivity due to high mobility of interlayer cations [6,7]. The majority of ion-exchangeable layered perovskite-like oxides can be converted into their protonated forms, which, besides being proton conductors [6,8] and photocatalysts for water splitting [9,10,11], exhibit the ability to intercalate water [10,11,12,13,14] and other molecules [15,16] and/or to form graft derivatives [16,17,18,19] susceptible to further exfoliation [10,20,21].

The hydrated form of HCa_2_Nb_3_O_10_ (usually referred to as HCa_2_Nb_3_O_10_·1.5H_2_O in the literature) belongs to the Dion-Jacobson phase and can be obtained from KCa_2_Nb_3_O_10_ oxide by ion-exchange in acid solutions [22]. It was shown that HCa_2_Nb_3_O_10_∙1.5H_2_O enables the intercalation of amines by an acid-base mechanism [23] and may be later exfoliated into nanolayers [24,25]. Both KCa_2_Nb_3_O_10_ and HCa_2_Nb_3_O_10_∙1.5H_2_O, as well as their exfoliated and restacked forms, exhibit photocatalytic properties [26,27,28]. Along with this form, there may be others with a lower water content. The ability to intercalate water molecules often plays a crucial role in other intercalation reactions and photocatalysis [9,29,30,31]. Hydrated protonated forms may comprise protons [13,30] or charged complexes like H^+^…*n*·H_2_O in their interlayer slab [32,33,34]. Obviously, water content and its state and localization should affect both the pathway and efficiency of chemical or photocatalytic reactions. From this perspective, an identification of proton-containing species and a comprehensive study of their motion in the interlayer slab is required.

Proton Nuclear Magnetic Resonance (NMR) is one of the most versatile experimental methods. It enables the identification of the proton-containing species and provides insight on the local structure [13,32,35,36,37] and information at the microscopic level on the dynamics of intercalated species [13,16,32,35,36,38]. In particular, by using ^1^H NMR, it was shown that both the local environment and the dynamics of hydrogen in these materials are affected by the stacking sequence of the perovskite-like slabs [39].

Here, we report on the results of the proton NMR spectroscopy and relaxation studies of the layered perovskite-like niobate HCa_2_Nb_3_O_10_ with different hydration levels: hydrated—α-form, dehydrated—*γ*-form, and intermediate—β-form. The details of their synthesis can be found in Section 3.

## 2. Results and Discussion

### 2.1. X-ray and TG Analysis

**Figure 1:**
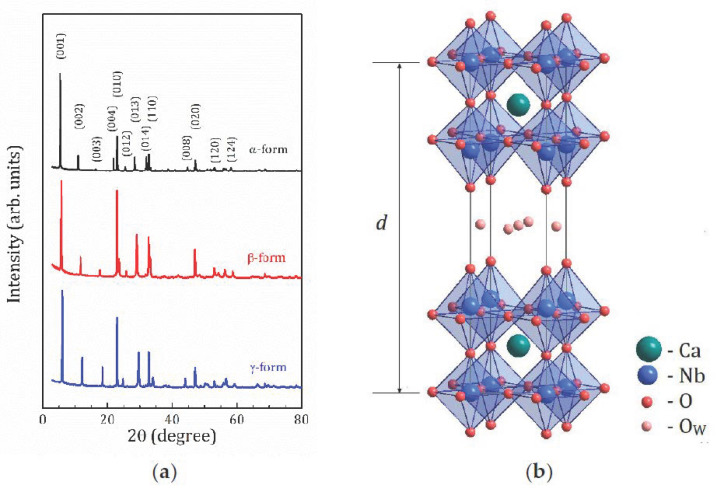
XRD powder patterns for the studied forms of HCa_2_Nb_3_O_10_·*y*H_2_O with different hydration levels (**a**) and the unit cell of HCa_2_Nb_3_O_10_·*y*H_2_O (**b**) with possible sites for the water oxygen O_w_.

Figure 1a shows the X-ray Diffraction (XRD) patterns of the studied HCa_2_Nb_3_O_10_·*y*H_2_O samples with different hydration levels. The XRD shows that the samples are practically monophase. All the samples can be described by the *P*4/*mmm* space group. The unit cell is shown in Figure 1b. The lattice parameters are listed in Table 1.

**Figure 2:**
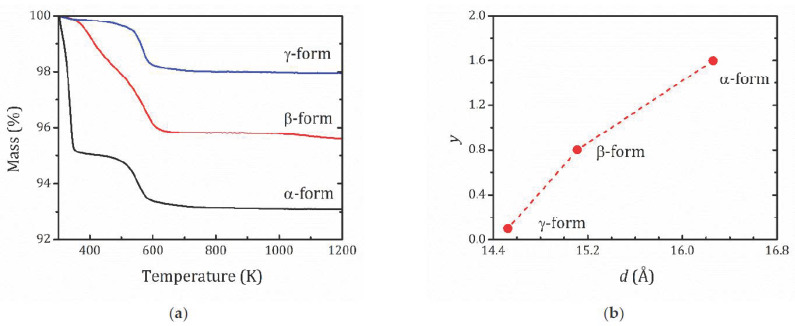
(**a**) TG curves for the studied forms of HCa_2_Nb_3_O_10_·*y*H_2_O with different hydration levels; (**b**) number of H_2_O molecules per formula units (*y*) versus the interlayer space (*d*).

The thermogravimetric (TG) curves that represent the mass decay due to the water release are shown in Figure 2a. As can be seen from the thermogravimetric curves, α-HCa_2_Nb_3_O_10_·*y*H_2_O exhibits behavior typical for a low-stable highly hydrated protonated form. Its thermal decomposition proceeds in two main steps, which is typical of protonated layered perovskite-like oxides [40,41,42]. The first step (T < 373 K) is associated with the release of the intercalated water and the formation of a dehydrated protonated compound:

HCa_2_Nb_3_O_10_·*y*H_2_O → HCa_2_Nb_3_O_10_ + *y*H_2_O. (1)

The second step of the mass loss that occurs at about 525 ÷ 550 K is related with thermal degradation, or so-called topochemical condensation:

HCa_2_Nb_3_O_10_ → Ca_2_Nb_3_O_9.5_ + 0.5H_2_O. (2)

The thermal decomposition of the γ-form demonstrates similar trends, but the mass loss at the first step is essentially low due to the much lower content of the intercalated water. Thermolysis of the β-form appears to be a more complex process, including gradual evolution of interlayer water in the temperature range of 373 ÷ 525 K, with the subsequent topochemical condensation of the protonated compound. The absence of the mass loss for the β-form at the beginning part of the TG curve indicates its greater thermal stability in comparison with the α-form.

According to the TG analysis, all the studied forms are fully protonated compounds with a substitution degree of K^+^ cations for protons H^+^ close to 100%. The water content as determined from TG curves results in 1.6, 0.8, and 0.1 H_2_O molecules per formula unit for α-, β-, and *γ*-forms, respectively. When describing layered structures, an important parameter is the interlayer distance *d*, the distance between the center of the adjacent perovskite slabs. For the studied structures, *d* = *c*; Figure 1b and Figure 2b show the correlation between the *d* parameter and the water content, which confirms that water molecules are located within the interlayer space.

### 2.2. ^1^H MAS NMR Study

**Figure 3:**
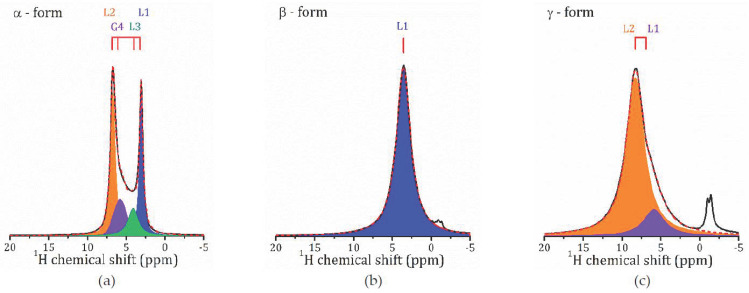
^1^H MAS NMR spectra in the α- (**a**), β- (**b**), and *γ*-forms (**c**) of HCa_2_Nb_3_O_10_·*y*H_2_O at 297 K.

Figure 3 shows the ^1^H MAS NMR spectra for the studied forms of HCa_2_Nb_3_O_10_·*y*H_2_O acquired at 259 K. As one can see, depending on the hydration level of HCa_2_Nb_3_O_10_·*y*H_2_O, the spectra differ from each other by the number of spectral lines, their position, and the linewidths. This shows the presence of different proton-containing species in the α-, β-, and *γ*-forms and their different mobilities.

At room temperature (297 K), the ^1^H spectrum of α-HCa_2_Nb_3_O_10_ (Figure 3a) consists of two narrow intense Lorentzian lines at 3.1 and 6.8 ppm, L1 and L2, respectively, and two lines of lower intensities: Lorentzian line L3 at about 4.1 ppm and Gaussian line G4 at 6.0 ppm. For β-HCa_2_Nb_3_O_10_ (Figure 3b), it consists of only one rather broad Lorentzian line at 3.6 ppm, whereas for *γ*-HCa_2_Nb_3_O_10_ (Figure 3c), the main signal is observed at 8.2 ppm (L2), with a shoulder at 5.9 ppm (L1) (a signal at about −2 ppm can be associated with surface defects and is not discussed further).

**Figure 4:**
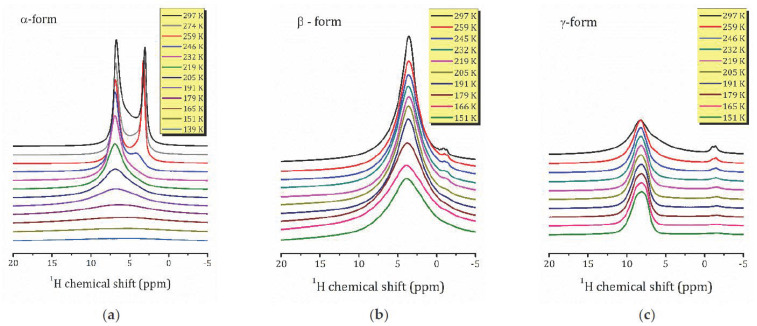
^1^H MAS NMR spectra in the α- (**a**), β- (**b**), and *γ*-forms (**c**) of HCa_2_Nb_3_O_10_·*y*H_2_O with the temperature decreasing.

**Figure 5:**
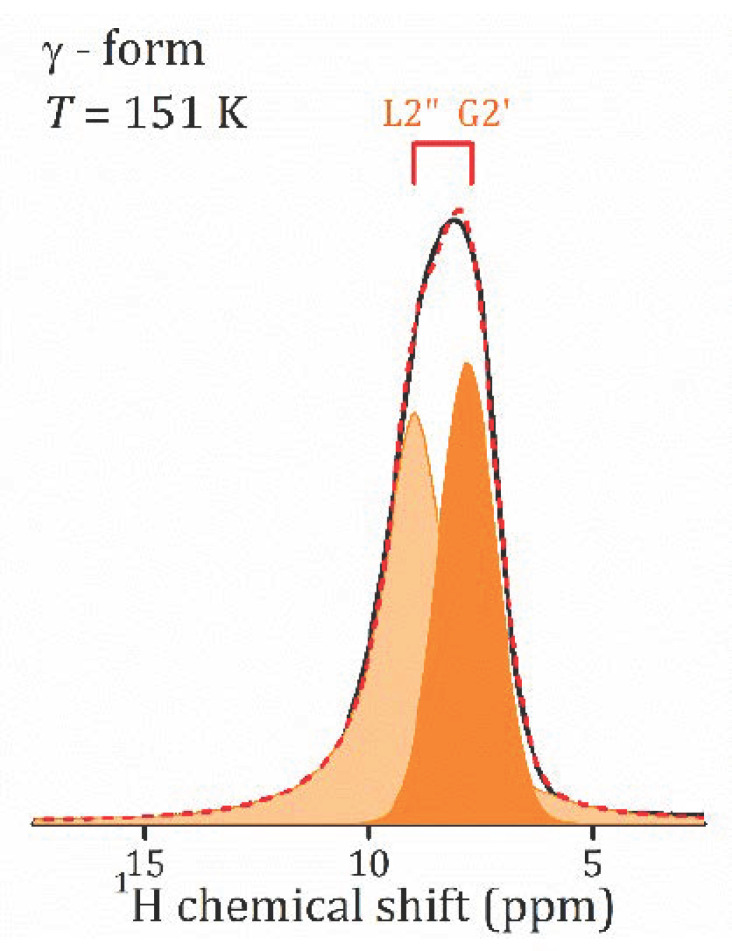
^1^H MAS NMR spectra at 151 K in *γ*-HCa_2_Nb_3_O_10_·*y*H_2_O and its decomposition.

**Figure 6:**
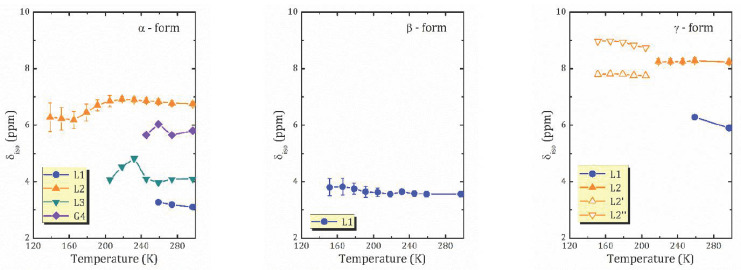

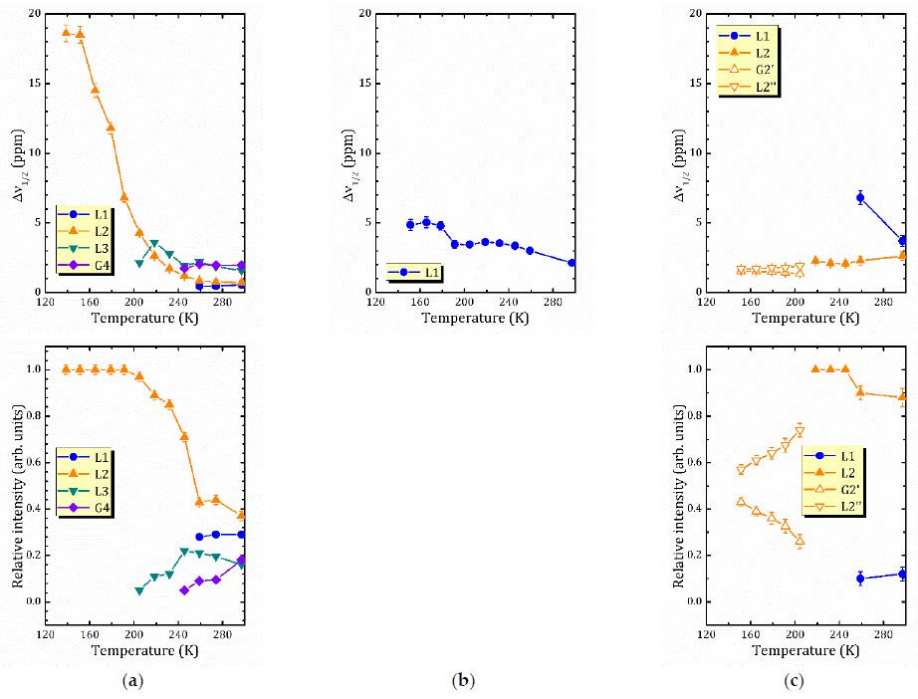
Temperature dependencies of the ^1^H isotropic chemical shift (the upper row), the line width at half maximum (the middle row), and the integral intensities (the bottom row) for the α- (**a**), β- (**b**), and *γ*- (**c**) forms of HCa_2_Nb_3_O_10_·*y*H_2_O.

To assign the spectral lines to the H-containing species, the evolution of the proton spectra with the temperature decreasing was studied; see Figure 4. As temperature decreases, the spectral lines broaden, and a redistribution of line intensities occurs. Let us first discuss the temperature evolution of the ^1^H MAS NMR spectrum of *γ*-form, HCa_2_Nb_3_O_10_·0.1H_2_O, which is characterized by the lowest water content. At room temperature, the contribution of the L2 line dominates, the relative intensity of L1 is of about 10%, and with sample cooling the line broadens and then disappears. Below 259 K, only the L2 line presents, and with the temperature further decreasing it splits into two lines: Lorentzian type at 8.9 ppm and Gaussian type at 7.8 ppm; see Figure 5. The temperature evolution of the spectral line parameters, namely the isotropic chemical shift (δ_iso_), the full width at half maximum (Δν_1/2_), and the relative integral intensities are shown in Figure 6a–c, respectively.

Based on the TG analysis, one can attribute L1 line to the bulk water. Normally its signal is expected at 5.5 ppm [22], but in a charged nanoconfinement it can be shifted towards a higher frequency. Its contribution is low with the temperature decreasing because of the slowing down of the molecular motion; thus, the line becomes too broad to be resolved. The most intensive line, L2, at about 8 ppm can be associated with the lattice protons in regular sites; e.g., in Ruddlesden–Popper phase H_2_La_2_Ti_3_O_10_·0.13H_2_O, the signal of isolated H^+^ was reported at 11–13 ppm [13,37]. The splitting of the line at low temperatures may point to two inequivalent cation positions. It is worth noting that down to 151 K, the linewidth of the spectral lines is almost unchanged. This indicates that within the studied temperature range, the proton mobility (translational diffusion) does not change significantly.

The ^1^H spectra of α-HCa_2_Nb_3_O_10_ exhibits the most dramatic changes with temperature: the high field part of the spectrum rapidly disappears with cooling; see Figure 4a. The temperature changes of the spectral parameters are plotted in Figure 6a. As one can see, with the temperature decreasing, the intensities of the spectral lines L1 and G1 rapidly drop, and after cooling down below 245 K, only L2 and L3 remain. Below 200 K, only the L2 peak is visible. Such a complex temperature behavior of the ^1^H MAS NMR spectrum of the α-form, as well as its structure, reflect (i) the variety of types of interlayer proton-containing species due to the high content of intercalated water in comparison with the other studied forms, and (ii) the non-obvious mechanisms of interaction between them in a charged environment. Interestingly, in α-form there is no signal associated with isolated protons. Moreover, despite a rather high water content, the only signal that can be associated with the bulk water, the line G4 at 6.1 ppm, has a very low intensity and, similar to the *γ*-form, rapidly disappears with cooling. Altogether, this suggests the presence of charged water complexed like H^+^…*x*H_2_O.

According to Ref. [43], the ^1^H chemical shift of H_3_O^+^ (*x* = 1), calculated for water solutions of mineral acids, is expected at 13.3 ppm. With *x* increasing, the ^1^H chemical shift decreases, e.g., for H^+^…*2*H_2_O it was predicted at 8.0 ppm. Our calculations carried out for isolated complexes give 7.3 and 4.6 (17.5) ppm for the isotropic chemical shift for free H_3_O^+^ and H_5_O_2_^+^ clusters, respectively (the number in parenthesis corresponds to the central proton). These calculations are supported by several experimental studies of hydrated layered oxides, in which the signal at 8–11 ppm was assigned to the H_3_O^+^ [32,36,44,45,46]. Hence, following both theoretical and experimental studies of other complex layered oxides, and accounting that for α-form of HCa_2_Nb_3_O_10_·*y*H_2_O there are 1.6 H_2_O molecules and one interlayer proton per one formula, it can be suggested that one water molecule participates in the formation of H_3_O^+^, the signal L2 at about 7 ppm, whereas other signals correspond to water molecules that are localized in different sites of the charged interlayer space or are part of the more extended charged complexes, like H^+^…*2*H_2_O.

The temperature behavior of the L2 linewidth, Figure 6a, is typical for solids [13,47,48] and reflects the slowing down of the molecular motion. Using the onset temperature of motional narrowing, *T*_MN_ = 150 K, one can estimate the activation energy of the line narrowing process within the semi-empirical Waugh-Fedin expression [49]:

(3) $$E_{a}\left(\mathrm{eV}\right)\;\approx \;1.61\;\times \;10^{-3}\;\cdot \;T_{\mathrm{MN}}\left(K\right).$$

This results in $E_{a}\approx$ 0.24(2) eV.

The ^1^H MAS NMR spectrum of the β-form consists of one line centered at about 3.6 ppm, which almost does not shift within the studied temperature range; see Figure 5. Taking into account that, according to the TG analysis, the β-form contains 0.8 H_2_O molecules per formula unit, and hence per interlayer cation H^+^, and no signal from H_3_O^+^ or H^+^ is observed, one can suppose that this line is the result of an exchange between the lattice protons (an expected signal at about 8 ppm as in the γ-form) and the non-hydrogen-bounded water (an expected signal at about 0.8 ppm).

### 2.3. ^1^H T_1ρ_ Study

To elucidate dynamic processes for all the studied compounds, the temperature dependencies of spin lattice relaxation times in the rotating frame, *T*_1ρ_ were measured. Relaxation measurements are more sensitive to changes in molecule dynamics than spectroscopic ones [47,50]. For the studied systems, the NMR relaxation is issued mainly by fluctuating strengths of ^1^H–^1^H dipole coupling. The latter, being dependent on the relative position of the interacting nuclear spins, is altered by motional processes. As a result, this leads to fluctuations of the Larmor frequency. This process can be described through a correlation function, $G\left(t\right)$:

(4) $$G\left(t\right)\;=\;\langle \Delta \omega \left(0\right)\;\cdot \;\Delta \omega \left(t\right)\rangle \;=\;G\left(0\right)\;\cdot \;g\left(t\right)$$

where the brackets represent the ensemble average; $g\left(t\right)$ contains information about dynamic processes, and its exact expression depends on the spin interaction and diffusion mechanism; $G\left(0\right)$ is determined by the mutual nuclear spin arrangement.

Commonly, to describe relaxation processes one uses a spectral relaxation function $j\left(\omega \right)$, which is a Fourier-transformed correlation function, $g\left(t\right)$. In terms of $j\left(\omega \right),$ the dipole contribution to NMR spin-lattice relaxation time $T_{1}$, a characteristic time for magnetization recovery after a perturbating pulse, can be written as follows:

(5) $$1/T_{1}=G\left(0\right)\;\cdot \;\left[\frac{1}{3}j\left(\omega_{0}\right)\;+\;\frac{4}{3}j\left(2\omega_{0}\right)\right],$$

where $\omega_{0}$ is the ^1^H NMR frequency. By analyzing the temperature dependence of spin lattice relaxation within an appropriate model, one can extract parameters of molecular motion, such as activation energies and correlation times. However, solids normally exhibit slower dynamics as compared to liquids. For such systems, the spin-locking technique is much more fruitful: by applying a locking field $\omega_{1}$, one can shift the minimum of the temperature dependence of the spin-lattice relaxation time towards the lower temperature in such a way that it falls within the measured temperature range [51,52,53,54,55]. More details can be found in Ref. [13]. At condition $\omega_{1}\ll \omega_{0}$, the relaxation time can be written as

(6) $$1/T_{1\rho }=G\left(0\right)\;\cdot \;\left[\frac{1}{2}j\left({2\omega }_{1}\right)\;+\;\frac{5}{6}j\left(\omega_{0}\right)\;+\;\frac{1}{3}j\left({2\omega }_{0}\right)\right].$$

For HCa_2_Nb_3_O_10_·*y*H_2_O, the upper limit of temperature is restricted by the water desorption, which according to the TG analysis (Figure 2a) occurs at *T* > 300 K. Application of the spin-locking technique helps to determine the spin motion parameters in a more accurate way.

**Figure 7:**
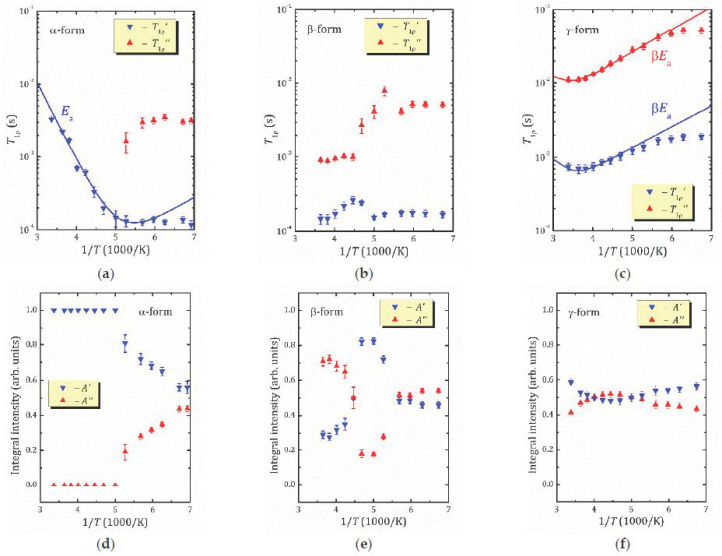
^1^H relaxation times *T*_1ρ_’, *T*_1ρ_’’ (**a**–**c**) and their relative contributions *A*’, *A*’’ to the magnetization recovery (**d**–**f**) for α- (**a**,**d**), β- (**b**,**e**), and *γ*- (**c**,**f**) forms of HCa_2_Nb_3_O_10_·*y*H_2_O versus inverse temperature. The solid lines show the fitting using the KWW correlation function.

**Figure 8:**
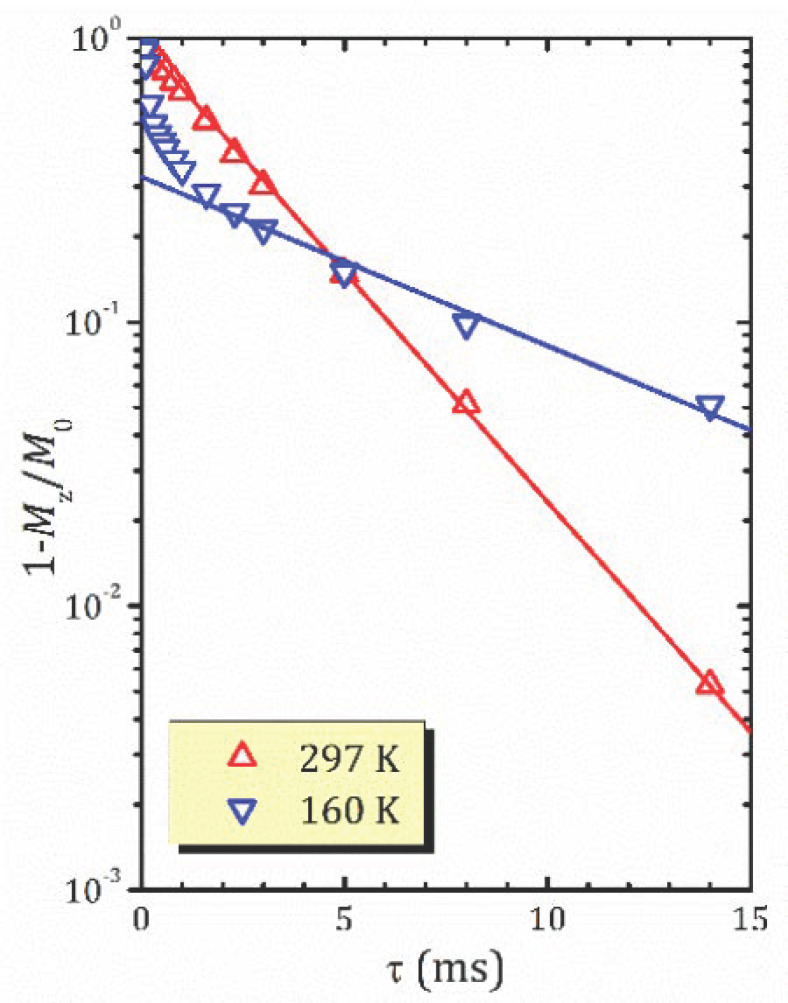
^1^H magnetization recovery curves for the α-form of HCa_2_Nb_3_O_10_·*y*H_2_O at 297 and 167 K with the exponential fit (solid lines); for 167 K the line corresponds to the slow component only.

The relaxation times *T*_1ρ_ for the studied forms of HCa_2_Nb_3_O_10_·*y*H_2_O plotted versus inverse temperature are shown in Figure 7a–c. It should be noted that for all the studied forms within the experimental temperature range, the magnetization recovery is mainly described by a two-exponential function, with characteristic spin-lattice relaxation times *T*_1ρ_’, *T*_1ρ_’’ differing from each other in one order of magnitude, except α-form, in which a mono-exponential behavior was observed above 200 K. Examples of the magnetization recovery curves (mono and two-exponential) are shown in Figure 8.

As it is clearly seen from Figure 7, depending on the hydration level, HCa_2_Nb_3_O_10_·*y*H_2_O demonstrates rather different *T*_1ρ_(1/*T*) behaviors. Let us first discuss the *γ*-form. The temperature dependence of *T*_1ρ_ for the least hydrated form of HCa_2_Nb_3_O_10_·*y*H_2_O exhibits features similar to H_1.83_K_0.17_La_2_Ti_3_O_10_·0.17H_2_O [13]. However, it should be noted that the applied locking field was not sufficient to displace the minimum in the middle of the studied temperature range. This complicates the analysis of the experimental data, but the higher pulse power would heat the system excessively.

To determine the proton motion parameters, we used the Kohlrausch-Williams-Watts (KWW) model [56,57,58] successfully applied to H_1.83_K_0.17_La_2_Ti_3_O_10_·0.17H_2_O [13]. Commonly, the relaxation in isotropic systems like liquids is described by the well-known Bloembergen-Purcell-Pound (BPP) model [59], which supposes that the exponential function $g\left(t\right)$ is as follows:

(7) $$g\left(t\right)\;=\;e^{-\left|t\right|/\tau_{c}}$$

and that the correlation time $\tau_{c}$ obeys the Arrhenius law: $\tau_{c}=\tau_{0}\exp\left(\frac{E_{a}}{k_{B}T}\right)$. Here $E_{a}$ is the activation energy of hydrogen motion, $k_{B}$ is the Boltzmann constant, and $\tau_{0}$ is a pre-exponential factor. The function in Equation (7) results in the following form of the spectral density:

(8) $$j\left(\omega \right)\;=\;\frac{2\tau_{c}}{1+{\left(\omega \tau_{c}\right)}^{2}}.$$

This model can be applied to solids, e.g., to describe the translational motion of hydrogen in metallic lattice, but requires some corrections to account for activation energy distribution [47,48], contribution of conduction electrons [60,61,62], and an exchange between different fractions [48,61,63].

In anisotropic systems, such as ionic conductors, the *T*_1_(1/*T*) dependence is asymmetric, and a stretched exponential KWW correlation function $g\left(t\right)\;=\;e^{-{\left(\left|t\right|/\tau_{s}\right)}^{\beta }}$ is more appropriate for its description. This means that the motion is correlated. These cooperative effects, similar to conduction electrons, contribute mainly at low temperatures, and the corresponding slope is reduced by β [55,56,57,58,64]. The spectral density function in this case can be written as

(9) $$j\left(\omega \right)\;=\;\frac{2\tau_{c}}{1+{\left(\omega \tau_{c}\right)}^{1+\beta }},$$

with the stretching exponent β ranging from 0 to 1.

As was mentioned above, due to the system limitations for the *γ*-form of HCa_2_Nb_3_O_10_·*y*H_2_O, one cannot observe the high temperature slope of the *T*_1ρ_(1/*T*) (Figure 7c), and hence one cannot determine correctly the stretching exponent. That is why to estimate the activation energy we used the parameter β = 0.28, as determined for H_1.83_K_0.17_La_2_Ti_3_O_10_·0.17H_2_O [13].

As seen from Figure 7c, the fast (*T*_1ρ_′) and slow (*T*_1ρ_″) components of *T*_1ρ_ exhibit very similar temperature dependencies. Moreover, within the studied temperature range, their contributions are almost tantamount. The fitting within the KWW model results in the very close values of $E_{a}$ and $\tau_{0}$: {${E_{a}}^{\prime }=0.223\left(2\right)$ eV, ${\tau_{0}}^{\prime }=8.8\left(5\right)\;\times \;10^{-10}$ s} and {${E_{a}}^{\prime \prime }=0.213\left(4\right)$ eV, ${\tau_{0}}^{\prime \prime }=7.8\left(3\right)\;\times \;10^{-10}$ s} for the slow and fast components, respectively. Accounting for the ^1^H MAS NMR data, one can suggest that these lines correspond to the isolated interlayer H^+^ ions or those in the vicinity of the water molecules.

For the most hydrated α-form above 200 K, the magnetization recovery is described by a single exponent, and the relaxation time *T*_1ρ_ rapidly decreases with temperature decreasing; see Figure 7a. However, as was mentioned above, below 200 K the character of the magnetization recovery changes, and a second exponent with a longer relaxation time appears. With further temperature decreases, the values of the both short (*T*_1ρ_′) and slow (*T*_1ρ_″) components do not change much; nevertheless, the contribution of the *T*_1ρ_″ component becomes more important and achieves about 44% at 145 K; see Figure 7d. It should be noted that, according to ^1^H MAS NMR spectra (Figure 5a), below 200 K there is only one spectral line at about 7 ppm. Such temperature dependencies of the both relaxation times and spectral parameters implicitly show the changes in dynamics of proton-containing species at 200 K. To estimate parameters of the proton motion in α-form, we applied the abovementioned KWW model to the high temperature branch of the *T*_1ρ_′(1/*T*). This results in the parameters $E_{a}=0.210\left(2\right)$ eV, $\tau_{0}=9.0\left(1\right)\;\times \;10^{-12}$ s, which can be associated with the translational motion of H_3_O^+^. Although above 200 K the magnetization recovery curve is described by a single exponential decay, ^1^H MAS NMR spectra exhibit the existence of different hydrogen-containing species in α-HCa_2_Nb_3_O_10_·*y*H_2_O (H_3_O^+^, H_2_O and other); see Figure 6a. This suggests a fast exchange between the species involved in the translational motion. Below 200 K, with the slowing down of the translation, other types of motion (e.g., reorientation) became more important.

The relaxation times for the β-form of HCa_2_Nb_3_O_10_·*y*H_2_O, Figure 7b, show a complex temperature dependence: within the studied temperature range there are at least two characteristic points (224 and 176 K) at which the proton dynamics change. These changes can also be observed in the temperature dependence of the proton linewidth, but they are less significant.

Therefore, the state of protons and water molecules located in the interlayer space, as well as their dynamics, are determined by the level of hydration. It is noteworthy that the formation of H_3_O^+^ is confirmed for the most hydrated α-form only, in which it is quite mobile even at low temperatures. For the γ-form, water molecules are not involved in the formation of hydronium ions; protons, behaving as lattice cations, can occupy at least two nonequivalent positions and participate in translational motion. The β-form is the most mysterious. The isotropic chemical shift of only the ^1^H spectral line indicates an exchange between different species, but it is not possible to unambiguously identify these species from the data obtained. The presence of two characteristic points on the temperature dependence of the proton relaxation time indicates that the mechanism of this exchange is temperature-dependent.

## 3. Materials and Methods

KCa_2_Nb_3_O_10_, a precursor for further synthesis of the protonic form, was synthesized by the standard ceramic method at normal conditions (air atmosphere and pressure) using CaO, Nb_2_O_5_, and K_2_CO_3_ as parent materials. CaO and Nb_2_O_5_ were taken in amounts to satisfy the stoichiometry of the reaction:

(10) $$4\mathrm{CaO}\;+3{\mathrm{Nb}}_{2}O_{5}+{\;K}_{2}{\mathrm{CO}}_{3}=2{\mathrm{KCa}}_{2}{\mathrm{Nb}}_{3}O_{10}+{\;\mathrm{CO}}_{2}↑\;.$$

To compensate for losses during calcination, potassium carbonate was taken with a 30% excess. All the components were mixed and ground in a planetary ball mill in n-heptane. The obtained powder was pelletized and then calcined at 800 °C for 12 h. After that, it was ground in an agate mortar, pelletized again, and calcined at 1100 °C for 24 h.

The first hydrated protonated form of KCa_2_Nb_3_O_10_, named as α-form of HCa_2_Nb_3_O_10_·*y*H_2_O, was prepared by acid processing of KCa_2_Nb_3_O_10_, with an excess of 12 M HNO_3_ (50 mL per 2.5 g of the oxide), at room temperature for 24 h. The product was centrifuged, washed with 50 mL of water three times to remove acid residues, and dried over CaO for 24 h. Subsequently, it was stored in a humid air atmosphere.

The second hydrated protonated form of KCa_2_Nb_3_O_10_, named as β-form of HCa_2_Nb_3_O_10_·*y*H_2_O, was prepared by hydrothermal treatment of the α- form. For this, 0.5 g of the latter was placed in the laboratory autoclave with 35 mL of water (volume filling approximately 80%) and processed at 150 °C for 7 d. The product obtained was centrifuged and dried over CaO for 24 h.

To obtain the dehydrated protonated form of KCa_2_Nb_3_O_10_, named as γ-form of HCa_2_Nb_3_O_10_·*y*H_2_O, the α-form was dried in a desiccator with a vacuum pump (about 1 × 10^−4^ atm) for 24 h.

Powder XRD analysis was done on a Rigaku Miniflex II diffractometer using monochromatic CuK_α_ radiation (λ = 0.154056 nm). Diffractograms were recorded in the 2*θ* range of 3–120° (step width 0.02°). The lattice parameters were calculated in the tetragonal system on the basis of all the reflections observed using DiffracPlus Topas 4.2 software.

TG analysis was carried out using a Netzsch TG 209 F1 Libra thermobalance. Analysis of samples was carried out in the temperature range 30–900 °C at a heating rate of 10 °C/min in an argon stream at a rate of 90 mL/min.

^1^H NMR experiments were done using a Bruker Avance IIITM 400 MHz solid-state NMR spectrometer (operating with Topspin version 3.2) using a double-resonance 4 mm low-temperature MAS probe. The temperature was changed within a temperature range of 139 to 297 K and controlled with an accuracy of 0.5 K. ^1^H NMR MAS spectra were recorded at rotor frequency 12 kHz. To measure *T*_1_ relaxation times, the spin-locking technique was applied, with the rf frequency of the locking pulse equal to 40 kHz. Tetramethylsilane (TMS) was used as an external standard.

The ^1^H magnetic shielding tensor for H_2_O, H_3_O^+^, and H_5_O_2_^+^ was calculated using the Gauge-Independent Atomic Orbital (GIAO) method [65] for the geometries optimized at the B3LYP/6-311G level of theory, as implemented in Gaussian 09 [66]. The theoretical isotropic chemical shift was estimated relative to the magnetic shielding tensor in TMS, calculated at the same level of theory.

## 4. Conclusions

The results of a comprehensive ^1^H NMR study of three different forms of the layered perovskite-like niobate HCa_2_Nb_3_O_10_·*y*H_2_O can be summarized as follows:

- For the most hydrated α-form, HCa_2_Nb_3_O_10_·1.6H_2_O, ^1^H MAS NMR spectra reveal the presence of different proton-containing species: H_3_O^+^, which comprises all the lattice protons (there is no signal that can be associated with H^+^ in regular sites), and water molecules that are localized in different sites of the interlayer slab and supposedly participate in the formation of charged complexes like H^+^…*2*H_2_O. With the temperature decreasing, the signal from the water proton is so broad that it is undetectable; the only detectable signal is from H_3_O^+^. The activation energy determined from the onset temperature of motional narrowing of 0.24(2) eV is in fair agreement with the relaxation data. Application of the KWW model with the stretching exponent β = 0.28 to the *T*_1ρ_(1/*T*) experimental dependence results in the following molecular motion parameters, which can be associated with the translational diffusion of H_3_O^+^: $E_{a}=0.210\left(2\right)$ eV, $\tau_{0}=9.0\left(1\right)\;\times \;10^{-12}$ s. Below 200 K, with the slowing down of the translational motion, other types of motion, such as reorientation, became more important.
- ^1^H MAS NMR spectrum of the β-form, HCa_2_Nb_3_O_10_·0.8H_2_O, within the studied temperature range consists of one line centered at about 3.6 ppm, which is the result of an exchange between lattice protons and the non-hydrogen-bounded water protons. The temperature dependence of the relaxation time evidences the presence of two characteristic points (224 and 176 K), at which proton dynamics changes.
- The proton NMR spectroscopy study of the γ-form, HCa_2_Nb_3_O_10_·0.1H_2_O, which is characterized by the lowest water content, reveals the presence of bulk water and interlayer H^+^ in two inequivalent positions. The temperature dependencies of the spin-lattice relaxation time in the rotating frame treated withing the KWW model with β = 0.28 results in the following parameters of the proton motion: $E_{a}=0.217\left(8\right)$ eV, $\tau_{0}=8.2\left(9\right)\;\times \;10^{-10}$ s.

We expect that the results obtained will clarify the relationship between the hydration level (and hence the type and, possibly, localization of hydrogen-containing species) and the photocatalytic activity of this class of layered materials. Currently, studies of the photocatalytic activity of the different forms of HCa_2_Nb_3_O_10_·*y*H_2_O are under evaluation.

## Figures and Tables

**Table 1:** Lattice parameters (*a*, *c*) and unit cell volume (*V*) for the studied forms of HCa_2_Nb_3_O_10_·*y*H_2_O.

Parameters	α-Form	β-Form	*γ*-Form
*a* (Å)	3.86517(3)	3.86550(9)	3.89267(8)
*c* (Å)	16.2627(2)	15.1125(7)	14.5254(4)
*V* (Å^3^)	242.957(6)	225.812(18)	220.102(14)

## Data Availability

The data presented in this study are available on request from the corresponding author.

## Sample Availability

Samples of the compounds are not available from the authors.
